# Global research trends in inflammaging from 2005 to 2024: a bibliometric analysis

**DOI:** 10.3389/fragi.2025.1554186

**Published:** 2025-04-10

**Authors:** Beier Jiang, Yi-ni Dong, Yu Xiong, Chun-xia Jiang, Jun Ping, Qi Wu, Liu-jun Xu, Run-zhe Shu, Dan-dan Gao, Sheng-mei Zhu, Wei-dong Ye, Feng Zhang

**Affiliations:** ^1^ Department of Respiratory and Critical Care, Quzhou Affiliated Hospital of Wenzhou Medical University, Quzhou, China; ^2^ The Joint Innovation Center for Engineering in Medicine, Quzhou Affiliated Hospital of Wenzhou Medical University, Quzhou, China; ^3^ Wenzhou Medical University, Wenzhou, China; ^4^ Shunxi Bio-Pharmaceutical Technology Co., LTD., Hangzhou, China

**Keywords:** inflammaging, immunosenescence, bibliometrics, research hotspots, scientific trends

## Abstract

**Background:**

Inflammaging, defined as chronic low-grade inflammation associated with aging, is considered a key factor in many age-related diseases. Despite growing research, comprehensive assessments of trends and focuses on this field over the past 2 decades remain lacking.

**Objective:**

To comprehensively analyze literature development trends, scientific priorities, and their evolution in the field of inflammaging from 2005 to 2024 using bibliometric analysis.

**Methods:**

Academic literature on inflammaging was retrieved from the Web of Science Core Collection. CiteSpace software was used as the bibliometric tool to analyze annual publication trends, contributing countries/regions, leading research institutions, primary journals, and keyword co-occurrence, including clustering and burst analysis in this field.

**Results:**

The study included 1,800 eligible articles, demonstrating a consistent growth in research publications over the past 20 years. The United States and Italy were the principal contributors. The University of Bologna had the highest publication. Professor Claudio Franceschi has been a leading figure in this field. Journal analysis shows that research themes predominantly focus on molecular biology/immunology and medicine/clinical fields. Keyword analysis identifies major research hotspots as “inflammaging,” “Crohn’s disease,” “periodontitis,” “immunosenescence,” “skeletal muscle,” “gut microbiota,” and “Parkinson’s disease.” Emerging term analysis indicates a shift from specific inflammatory diseases to broader aging and immune modulation studies.

**Conclusion:**

This first systematic assessment of literature trends in the field of inflammaging from 2005 to 2024 reveals sustained academic growth and an increasingly deep research focus.

## Highlights

• This study provides the first systematic, bibliometric assessment of inflammaging research trends over 20 years, offering a comprehensive overview that identifies global changes and developments.
 • The study highlights the interdisciplinary nature of inflammaging research, encompassing molecular biology, immunology, and clinical applications, illustrating the complex interplay between aging and chronic inflammation.
 • By analyzing contributions from various countries and institutions, this study underscores the global scope of research and the importance of international collaborations in advancing inflammaging understanding.
 • This study reviews past and current research and forecasts future directions, emphasizing emerging research hotspots and the need for more comprehensive studies on inflammaging’s systemic and molecular mechanisms.

## 1 Introduction

Inflammaging, a chronic, low-grade inflammation that occurs with aging, is widely recognized as a major driver of age-related disease progression (Giunta et al., 2022; Dugan et al., 2023). In contrast to acute inflammation, inflammaging is characterized by a gradual increase in inflammatory factors, immune system imbalance, and prolonged inflammatory signaling, often accompanied by immune senescence (Walker et al., 2022). This chronic inflammation marks and accelerates aging and functional decline, posing a serious health threat to the elderly (Teissier et al., 2022; Arosio et al., 2023).

The relationship between inflammaging and the development of various diseases is increasingly evident. For instance, inflammaging drives cardiovascular diseases like atherosclerosis by persistently elevating pro-inflammatory factors such as CRP and IL-6, which cause endothelial damage and lipid deposition (He et al., 2022; A and M, 2022). In neurodegenerative conditions like Alzheimer’s and Parkinson’s, inflammaging worsens neuronal damage by continuously activating microglial cells and releasing cytokines like TNF-α (Dias-Carvalho et al., 2024; Khan et al., 2024). In cancer, inflammaging fosters uncontrolled cell proliferation and tumor aggressiveness through a chronic inflammatory environment that continually activates NF-κB and elevates IL-1α, enhancing tumor immune suppression and affecting cancer progression (M et al., 2024; Olivieri et al., 2013). Thus, as a key biological marker of aging, inflammaging is essential for understanding and addressing age-related diseases, providing significant clinical insights for slowing aging and preventing related diseases.

In recent years, research on inflammaging, from molecular mechanisms to disease therapies, has gradually increased globally. Researchers are analyzing changes in immune cell function and the dynamic expression of pro-inflammatory factors during aging to uncover the molecular basis of inflammaging. For instance, the free radical theory posits that oxidative stress-induced inflammation drives aging (Pagan et al., 2022; Sobhon et al., 2023); telomere shortening contributes to cell division limits and inflammation (Nga et al., 2024); and cellular senescence fosters systemic inflammation through senescence-associated secretory phenotype (SASP) secretion (Chin et al., 2023). Research into intervention strategies, including anti-inflammatory drugs, dietary changes, exercise, and therapies targeting cellular senescence, has shown promising progress, offering potential solutions to mitigate inflammaging (Chen et al., 2020; Wawrzyniak-Gramacka et al., 2021; Ding and Xu, 2021). Recently, AI-based predictive models have also emerged as innovative tools to assess the risk and predict the onset of inflammaging, enabling more personalized approaches to interventions (Zhou et al., 2024; Xu et al., 2024). Despite these advances, a comprehensive quantitative assessment of global research trends, hotspots, and future directions has not yet been conducted, which limits our ability to effectively translate research findings into clinical practice. Without this understanding, it is difficult to prioritize the most relevant interventions and guide clinical decision-making, highlighting the need for further scientific efforts to address these knowledge gaps.

In this context, bibliometrics is an essential tool for understanding global research trends in inflammaging. Unlike traditional research methods such as meta-analyses or randomized controlled trials (RCTs), which focus on synthesizing specific findings or clinical outcomes, bibliometric analysis provides a broader view of the research landscape (Linnenluecke et al., 2020). It examines the quantity, quality, and evolution of scientific publications over time, helping us identify emerging research topics, track shifts in research priorities, and highlight influential studies that may not yet be well-known. Previous bibliometric analyses (Hu et al., 2024; D’Andria Ursoleo et al., 2024) have demonstrated the widespread application in identifying research trends and hotspots across multiple disciplines, and our study extends these efforts to inflammaging. By conducting a worldwide quantitative literature analysis on inflammaging, this study aims to identify and validate hot topics, research frontiers, and future directions in aging research. It is worth noting that citation counts of papers are not only influenced by their relevance but also by the time factor. Newer publications may initially have fewer citations due to having been published more recently. Therefore, when interpreting bibliometric data to better understand the development of the field, we simultaneously considered the publication time. This research will advance scientific understanding of inflammaging and improve strategies for addressing the challenges of an aging society.

## 2 Methods

### 2.1 Data collection

This study employed the Web of Science Core Collection (WoSCC), including the Social Sciences Citation Index (SSCI) and the Science Citation Index Expanded (SCI-Expanded), as the primary literature source. Literature research was conducted through 14 November 2024, using a Topic Search (TS) that encompassed article titles, abstracts, and keywords. Literature selection criteria included: 1) Publication date range from January 2005 to December 2024; 2) Focus on inflammaging; 3) Publications in English; 4) Document types limited to articles and review articles. Preprints, conference papers, and other non-peer-reviewed documents were excluded to ensure the quality and reliability of the included studies. The search keywords strategy was TS = (“Inflammaging” OR “inflamm aging” OR “inflamm-aging”), yielding 1,620, 470, and 369 documents, respectively.

### 2.2 Data analysis

Data analysis utilized detailed information and complete citation records from articles, exported from WoSCC in TXT and BIB formats. These data were imported into CiteSpace software to create scientific knowledge maps. A time-slicing technique was used to analyze inflammaging research dynamics from January 2005 to December 2024, treating each year as an independent unit. The cosine algorithm in CiteSpace calculated the strength of node associations, analyzing the top 50 most frequent words in each time slice. Maps were optimized with the pathfinder algorithm and pruning of sliced networks. Network nodes were designated as research institutions and keywords, with visualization maps created using the minimum spanning tree algorithm to reveal the evolution of research themes over time. To assess the impact of research, we considered the absolute citation index. Although alternative metrics, such as Field-Weighted Citation Impact (FWCI) (D’Andria Ursoleo et al., 2024), exist, we chose not to include them. Absolute citation counts are more commonly used in bibliometric studies and provide a simpler basis for cross-study comparisons. Additionally, to identify emerging research themes, burst detection algorithms proposed by Goldberg et al. were utilized (Goldberg et al., 2002).

The present study was performed in accordance with the methodology employed by previously published bibliometric analyses (D’Andria Ursoleo et al., 2024; D’Andria Ursoleo et al., 2025a; D’Andria Ursoleo et al., 2025b), which have been widely adopted across various scientific specialties. Furthermore, the study was conducted following the guidelines of the BIBLIO checklist for bibliometric studies, ensuring methodological rigor and completeness.

### 2.3 Tools

Data analysis primarily utilized CiteSpace 6.4. R1 Basic, developed by Dr. Chaomei Chen at Drexel University. CiteSpace is a powerful tool for co-occurrence and co-citation analysis and has been widely used in bibliometric studies to visualize scientific research trends. The selection of CiteSpace was based on its robust ability to generate meaningful visualizations, identify research frontiers, and analyze citation networks. All data plotting and statistical analysis were conducted with GraphPad Prism 10.1.2. This tool is a widely recognized software in scientific research for its reliability in ensuring result accuracy and statistical rigor, making it a preferred choice for data analysis in this study. Additionally, the “biblioshiny” R package was used to visualize Lotka’s Law and Bradford’s Law, assessing the productivity of scientific authors and the concentration of literature.

## 3 Results

### 3.1 Publication trend analysis

**FIGURE 1:**
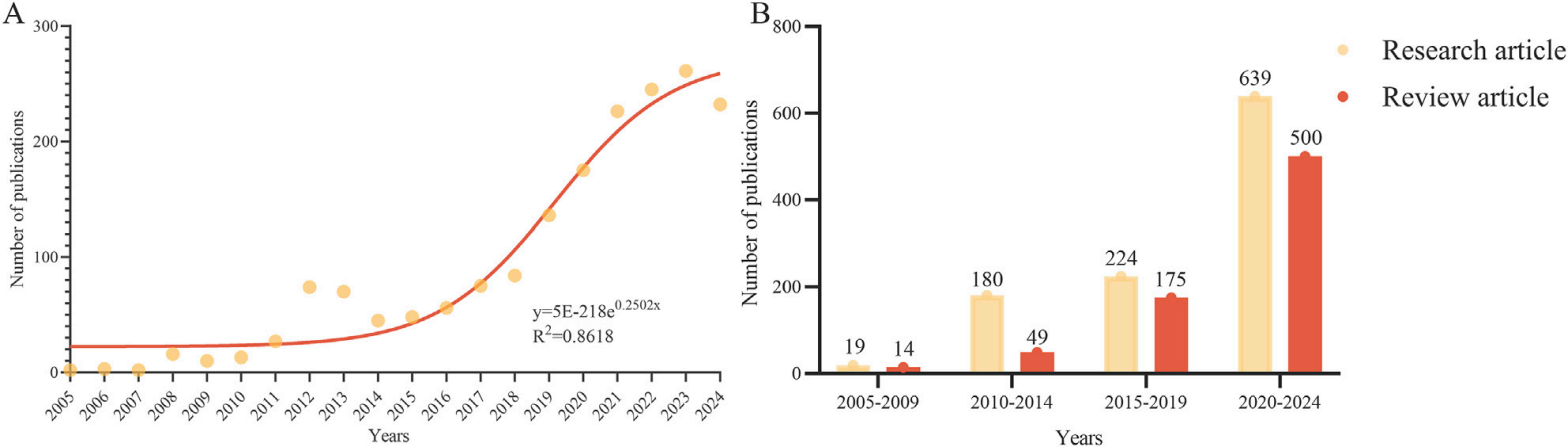
Publications analysis. **(A)** Trends of Inflammaging (2005-2024). **(B)** Distribution of research and review articles.

From 2005 to 2024, a total of 1,800 articles were identified, consisting of 1,062 articles and 738 review articles. The volume of literature published during this period showed an overall upward trend (Figure 1A). Specifically, the publication volume was relatively low from 2005 to 2010. In 2012, there was a significant increase in the number of published studies, which was followed by a brief decline. From 2015, the publication pace accelerated again, averaging over 150 papers annually. Using the exponential growth model y = 5E−218e^0.2502x^, projections indicate that the number of published studies will approach 300 by 2024, demonstrating a high fit with the actual data (*R* ^2^ = 0.8618). The ratio of research articles to review articles, calculated in 5-year intervals from 2005 to 2024, demonstrates that research articles have consistently outnumbered review articles (2005–2009: 1.36, 2010–2014: 3.57, 2015–2019: 1.28, 2020–2024: 1.28). Notably, during 2010–2014, this ratio peaked at 3.57, indicating a period of intensified research activity and an increased demand for new knowledge generation (Figure 1B).

### 3.2 Country/region analysis

The 1,800 publications of inflammaging were contributed by 84 different countries/regions. The top 10 contributing countries/regions are listed in Table 1. The United States leads with 534 papers and a centrality of 0.26, underscoring its leadership in global inflammaging research. Despite fewer articles than the United States, Italy exhibits the highest centrality at 0.30, demonstrating significant influence and extensive international collaboration in inflammaging research. China, with 187 papers, ranks third yet shows low centrality (0.02), suggesting room for improved international collaboration. Other contributing countries include Germany, the United Kingdom, Spain, France, Canada, the Netherlands, and Russia.

**TABLE 1:** Top 10 countries/regions.

Rank	Country	Centrality	Count
1	United States	0.26	534
2	Italy	0.30	373
3	China	0.02	187
4	Germany	0.09	179
5	England	0.27	148
6	Spain	0.13	128
7	France	0.04	90
8	Canada	0.10	86
9	Netherlands	0.09	68
10	Russia	0.07	61

The network map further reveals international collaboration patterns (n = 84, E = 618, density = 0.1773, Figure 2). Within the network, the United States, United Kingdom, Spain, and Italy show prominent centrality, visually highlighted by pink circles. This suggests that these countries not only contribute a substantial number of publications but also play a pivotal role in shaping the direction of inflammaging research. Network density measures the connectivity between nodes as the ratio of actual to possible connections, typically ranging from 0 to 1. The network density of 0.1773 indicates moderate connectivity between countries, suggesting that while there is significant international collaboration, the overall connectivity remains relatively low. This highlights an opportunity for enhancing global cooperation, especially among countries with lower centrality.

**FIGURE 2:**
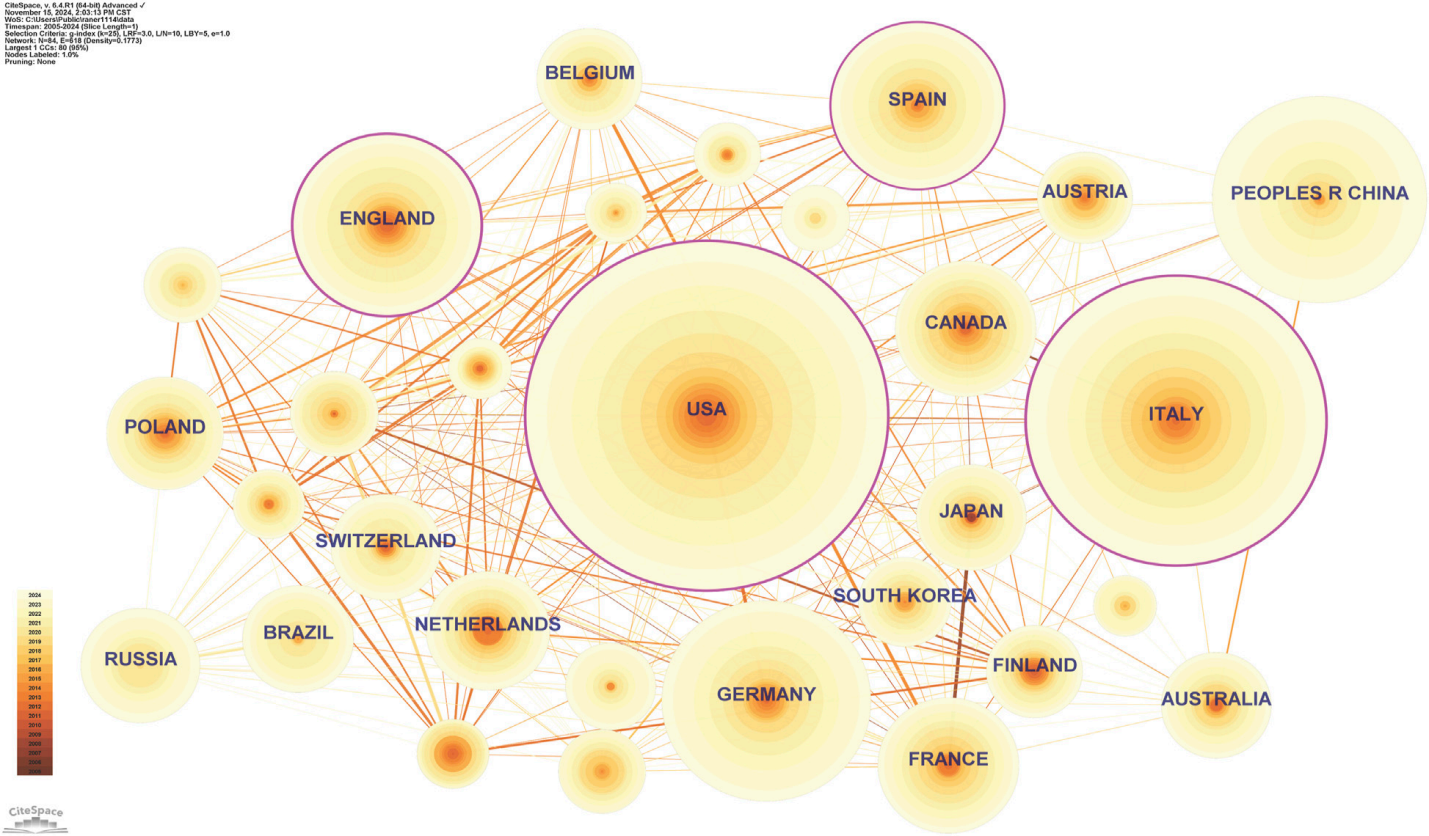
Country/region contribution and collaboration network.

### 3.3 Author Analysis

**FIGURE 3:**
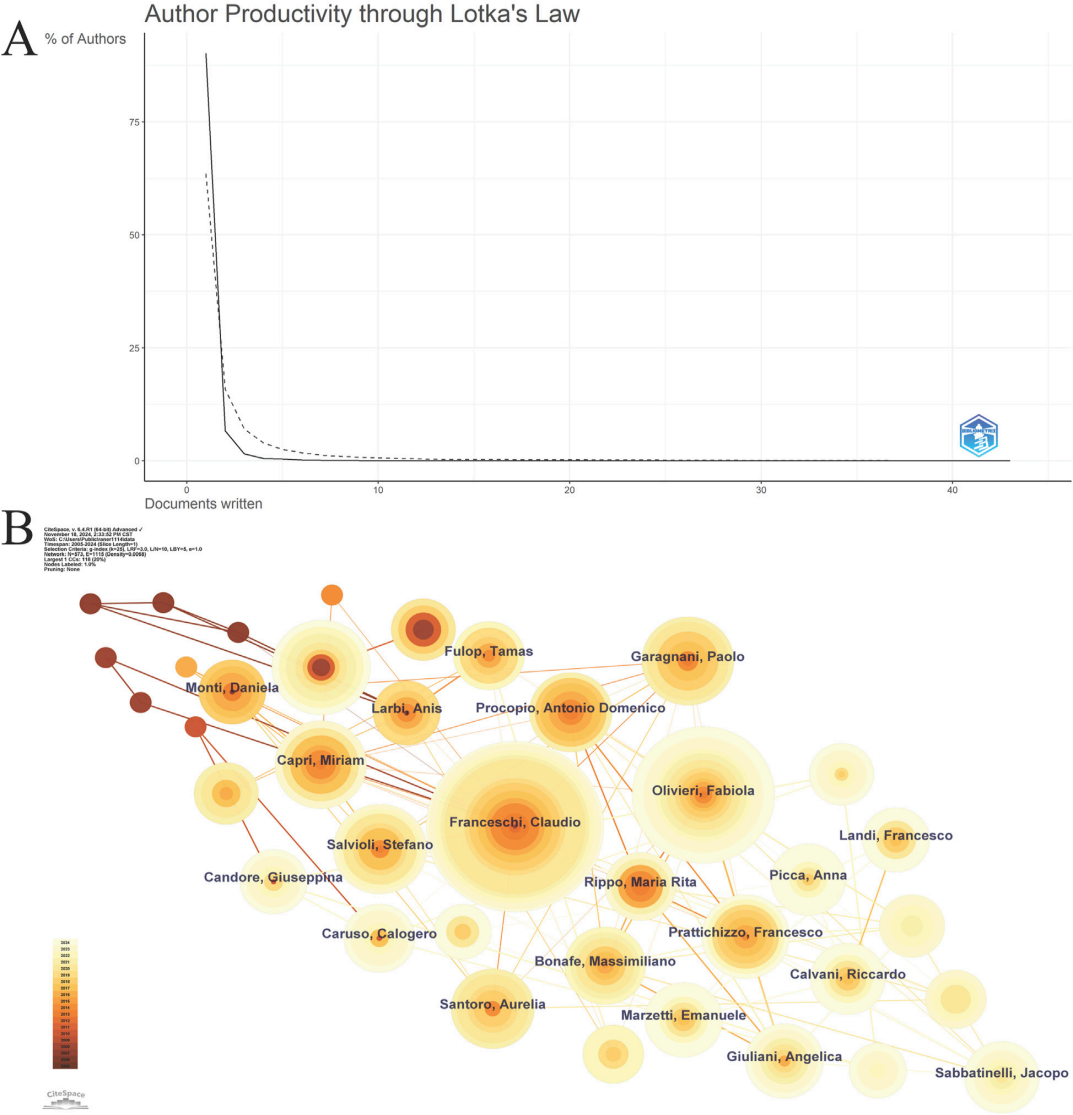
Author analysis **(A)**. Lotka’s law view; **(B)**. Author collaboration network.

A total of 573 authors have contributed to inflammaging. Overall, author productivity in this field adheres to Lotka’s Law, which states that few authors are highly prolific, while most have fewer publications (Figure 3A). Among the top ten authors, Italian researchers, notably Professor Claudio Franceschi with 69 papers, play a significant role in these scientific activities (Table 2). The author research network (n = 573, E = 1,115, density = 0.0068, Figure 3B) reveals a relatively dispersed collaboration network, indicating sparse collaborative relationships.

**TABLE 2:** Top 10 authors.

Rank	Author	Institution	Country	Count
1	Franceschi, Claudio	IRCCS Istituto delle Scienze Neurologiche di Bologna (ISNB)	Italy	69
2	Olivieri, Fabiola	Marche Polytechnic University	Italy	44
3	Salminen, Antero	University of Eastern Finland	Finland	21
4	Salvioli, Stefano	University of Bologna	Italy	19
5	Capri, Miriam	University of Bologna	Italy	18
6	Garagnani, Paolo	University of Bologna	Italy	18
7	Prattichizzo, Francesco	IRCCS Multimedica	Italy	17
8	Procopio, Antonio Domenico	IRCCS INRCA	Italy	15
9	Santoro, Aurelia	University of Bologna	Italy	15
10	Bonafe, Massimiliano	IRCCS Azienda Osped Univ Bologna	Italy	14

### 3.4 Institution analysis

**FIGURE 4:**
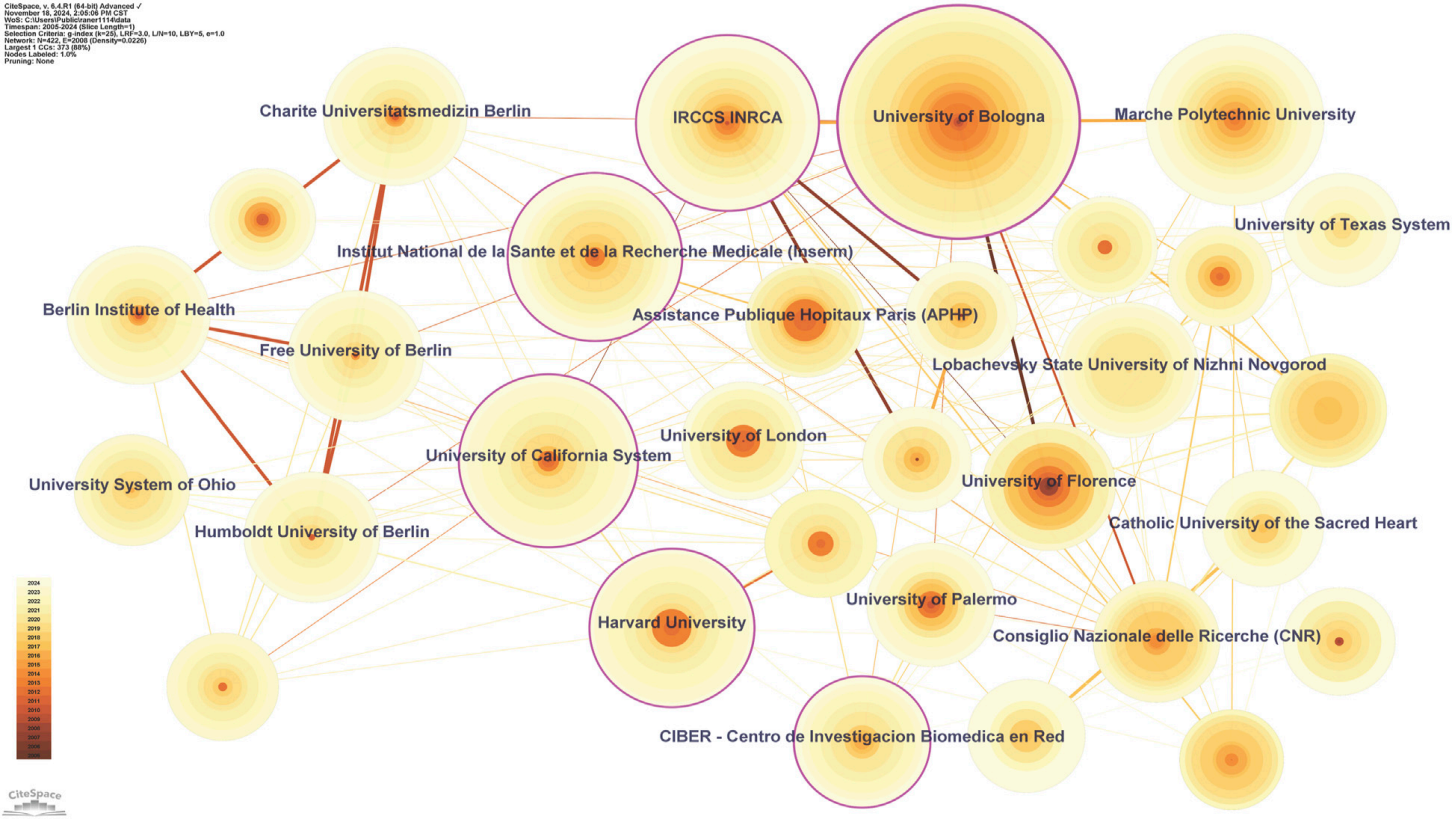
Institutional collaboration network.

Globally, 422 institutions have contributed to the literature in inflammaging, with the top ten institution listed in Table 3. The University of Bologna, with 95 published papers and a centrality of 0.21, leads these institutions, underscoring its pivotal role in this research area. Following closely are IRCCS INRCA with 53 papers (centrality of 0.13) and the University of California System with 50 papers (centrality of 0.18). The institutional research network map (n = 422, E = 2008, density = 0.0226, Figure 4) shows that 422 institutions are involved in 2008 cooperative relationships. However, the low network density (0.0226) suggests that, while many institutions are engaged in research, the collaborations are spread across a wide range of institutions rather than concentrated in a few core institutions. This indicates the potential for more intensive and targeted collaboration among institutions to enhance research impact.

**TABLE 3:** Top 10 institutions.

Rank	Institution	Country	Centrality	Publications
1	University of Bologna	Italy	0.21	95
2	IRCCS INRCA	Italy	0.13	53
3	University of California System	United States	0.18	50
4	Marche Polytechnic University	Italy	0.03	50
5	Institut National de la Sante et de la Recherche Medicale (Inserm)	France	0.17	48
6	Harvard University	United States	0.13	42
7	Charite Universitatsmedizin Berlin	Germany	0.04	32
8	Berlin Institute of Health	Germany	0.04	32
9	Lobachevsky State University of Nizhni Novgorod	Russia	0.07	31
10	Free University of Berlin	Germany	0.01	30

### 3.5 Journal Analysis

**FIGURE 5:**
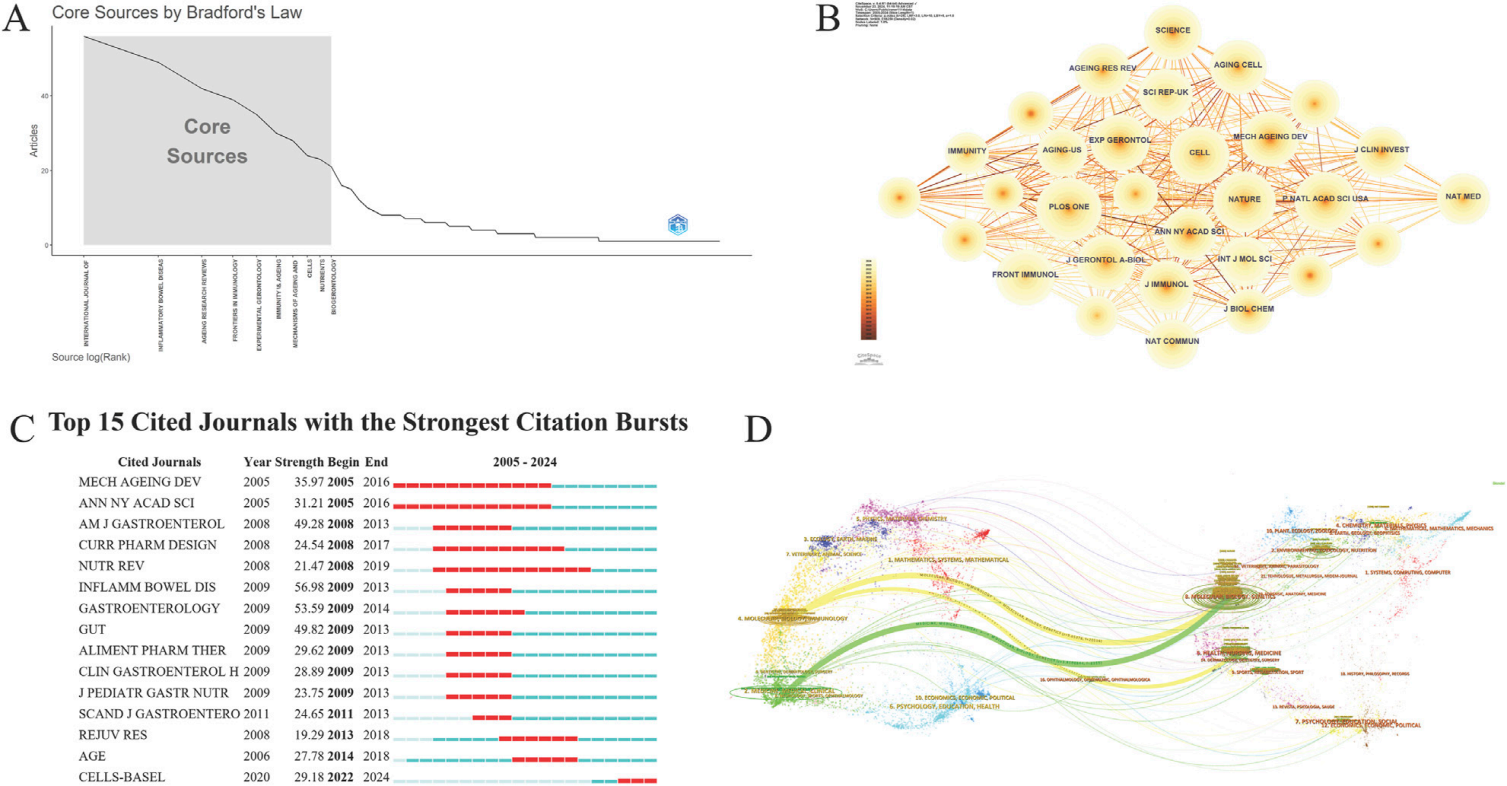
Journal analysis **(A)**. Bradford’s law view; **(B)**. Journal collaboration network; **(C)**. Cited journals burst view; **(D)**. Dual overlay map of journals.

The literature is published across 909 journals, with the top ten detailed in Table 4. “Inflammatory Bowel Diseases” leads with 98 articles, representing 5.40% of total publications. It is closely followed by the “International Journal of Molecular Sciences” with 97 publications and “Frontiers in Immunology” with 73. Additionally, “Ageing Research Reviews,” with an impact factor of 12.5, holds a leading position, indicating high academic influence. The Bradford curve reveals a concentration of research in a few high-impact journals, indicating a trend toward publication centralization (Figure 5A). Despite the large number of journals, the collaboration network diagram shows low density (n = 909, E = 8,259, density = 0.02), indicating dispersed citations and collaborations (Figure 5B).

**TABLE 4:** Top 10 journals.

Rank	Journals	Publications	Percentage	If
1	Inflammatory Bowel Diseases	98	5.40%	4.5
2	International Journal of Molecular Sciences	97	5.38%	4.9
3	Frontiers in Immunology	73	4.06%	5.7
4	Ageing Research Reviews	67	3.72%	12.5
5	Experimental Gerontology	57	3.17%	3.3
6	Immunity and Ageing	55	3.06%	5.2
7	Nutrients	43	2.39%	4.8
8	Mechanisms of Ageing and Development	40	2.22%	5.3
9	Cells	39	2.17%	5.1
10	Aging Cell	34	1.89%	8.0

Note: IF (Impact Factor) statistics are as of 26 November 2024.

The top ten most-cited journals, listed in Table 5, show “PLOS ONE” leading with 1,110 citations, though its average citation per article (123.3) is lower than “Nature,” which garnered 1,062 citations from just one article, reflecting high quality. Despite publishing fewer articles, “Cell” and “Science” have high citation counts per article, underscoring their significant impact within the scientific community. The burst view indicates a notable increase in citations for “CELLS-BASEL” from 2022 to 2024 (Figure 5C). Overlay maps show that research journals are concentrated, with citations clustered mainly in Molecular/Biology/Immunology and Medicine/Medical/Clinical fields, generating citation chains within these areas (Figure 5D).

**TABLE 5:** Top 10 cited journals.

Rank	Journal	Publications	Centrality	Citations	If
1	Plos One	9	0.01	1,110	2.9
2	Nature	1	0.02	1,062	50.5
3	Proceedings of The National Academy of Sciences of The United States of America	3	0.02	1,001	9.4
4	Cell	39	0.02	942	45.5
5	Experimental Gerontology	57	0.01	932	3.3
6	Journals of Gerontology Series A-Biological Sciences and Medical Sciences	22	0.01	896	4.3
7	Mechanisms of Ageing and Development	40	0.01	886	5.3
8	Frontiers in Immunology	73	0.03	873	5.7
9	Aging Cell	34	0.03	868	8.0
10	Science	3	0.02	856	44.7

Note: IF (Impact Factor) statistics are as of 26 November 2024.

### 3.6 Keyword Analysis

**FIGURE 6:**
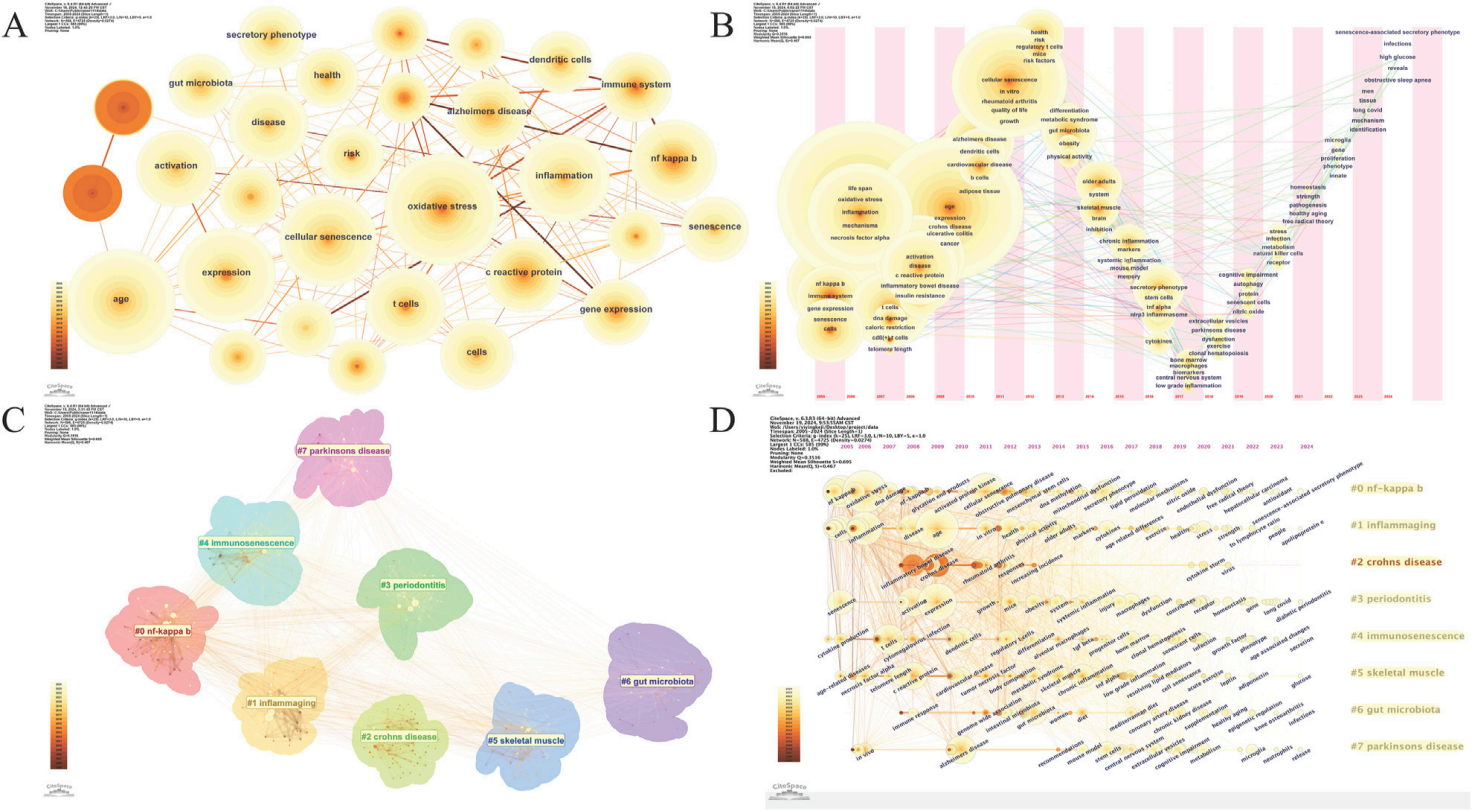
Keyword Analysis **(A)**. Keyword Co-occurrence Network; **(B)**. Keyword Time zone view; **(C)**. Keyword Cluster view; **(D)**. Timeline View of Keyword Clusters.

A total of 588 keywords were identified. The ten most frequently occurring keywords were listed in Table 6. “Oxidative stress,” the top keyword, appeared 266 times and has a centrality of 0.07, highlighting its pivotal role in inflammaging research. “Age” follows closely, appearing 238 times with a centrality of 0.04, underscoring its significance in inflammaging studies. Other significant keywords are “expression” (195 times), “cellular senescence” (186 times), and “inflammation” (170 times), emphasizing the relevance of gene expression and cellular aging in chronic inflammation. “Activation” and “nf kappa b” appeared 145 and 139 times, respectively, reflecting the crucial role of these signaling pathways in inflammatory responses. The keyword “t cells” also featured prominently, appearing 128 times, indicating its focus in research. “Disease” appeared 124 times and “c reactive protein,” a marker of inflammation, 119 times, underscoring their central roles in studying inflammaging mechanisms. A keyword co-occurrence map (n = 588, E = 4,725, density = 0.0274, Figure 6A) shows the dispersed relationships among keywords. The network density of 0.0274 suggests that while there are meaningful connections between keywords, these connections are relatively sparse, indicating that the research community is exploring multiple areas without highly concentrated collaboration in any single topic.

**TABLE 6:** Top 10 keywords.

Rank	Keyword	Centrality	Count
1	oxidative stress	0.07	266
2	age	0.04	238
3	expression	0.03	195
4	cellular senescence	0.04	186
5	inflammation	0.07	170
6	activation	0.05	145
7	nf kappa b	0.03	139
8	t cells	0.06	128
9	disease	0.04	124
10	c reactive protein	0.05	119

A keyword timeline view clearly displays the evolution of high-frequency keywords (Figure 6B). As time progresses, new research areas emerge, with keywords like “long covid,” “microglia,” and “cognitive impairment” becoming prominent in later years. This indicates a shift toward the study of neuroinflammation, cognitive decline, and the long-term effects of infections on aging, reflecting a broader expansion of research themes. Clustering analysis of the 588 keywords using the log-likelihood ratio algorithm identified eight major clusters, representing key research directions in inflammaging (Figure 6C). The clusters have an average silhouette value of 0.695, exceeding the standard of 0.5, indicating high consistency and compactness. Additionally, a Q-value of 0.3516, above the threshold of 0.3, validates the significance of the community structures in the clustering analysis. The largest cluster (#0) is “nf kappa b,” followed by: #1“inflammaging,” #2“crohns disease,” #3“periodontitis,” #4“immunosenescence,” #5“skeletal muscle,” #6“gut microbiota,” #7“parkinsons disease.” The interrelations and keywords within each cluster are visually represented in a timeline view (Figure 6D).

### 3.7 Keyword Burst analysis

**FIGURE 7:**
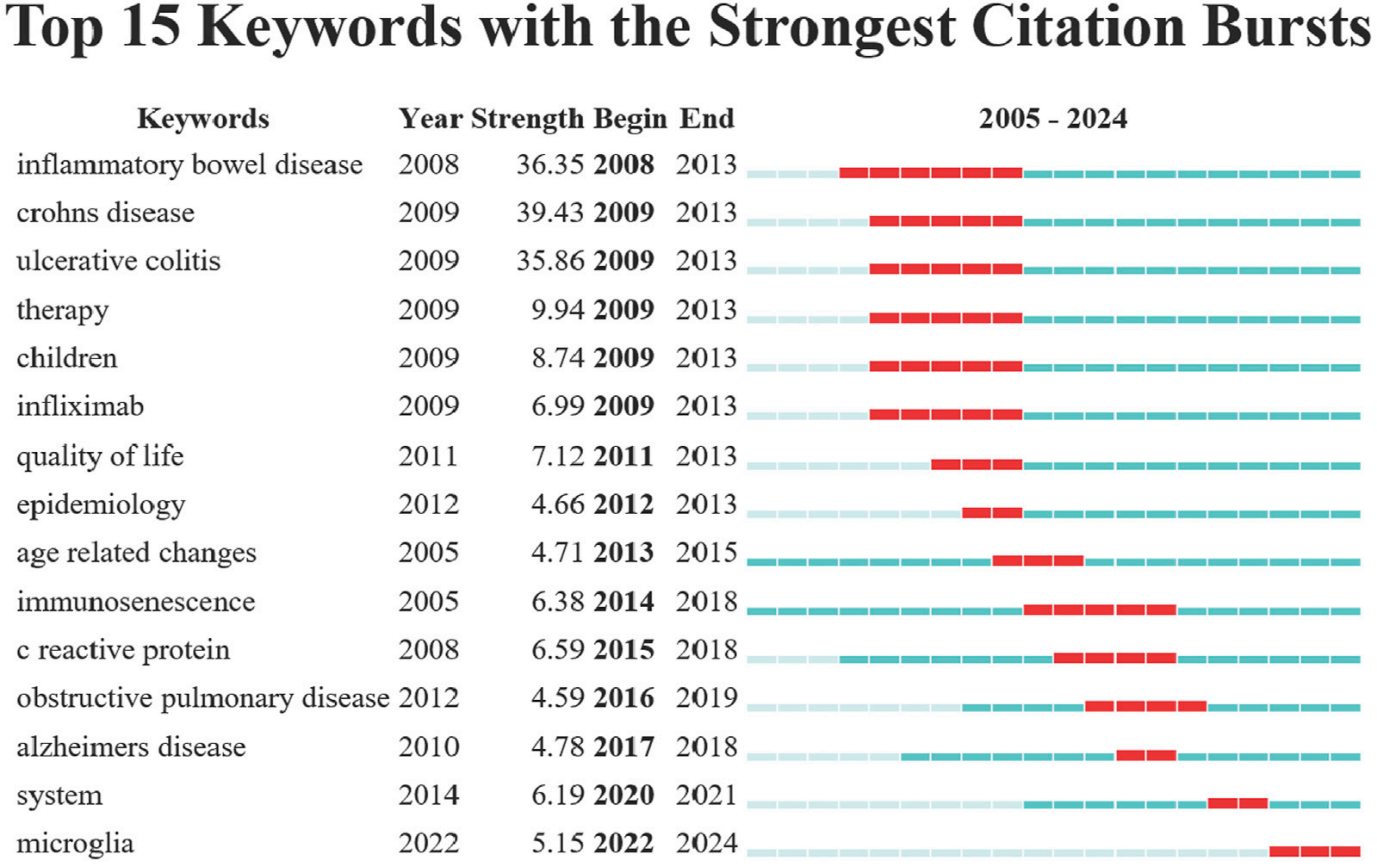
Keyword Burst view.

Burst analysis identified 15 keywords with the strongest citation bursts (Figure 7). These keywords highlight research hotspots in inflammaging over various periods. From 2008 to 2013, “inflammatory bowel disease,” “Crohn’s disease,” and “ulcerative colitis” showed strong citation bursts (average strength = 37.21), likely due to advancements in their treatment and diagnosis. From 2014 to 2021, “immunosenescence” and “C Reactive Protein” experienced strong citation bursts, highlighting their emergence as focal points in research on immune aging and inflammation. From 2022 to 2024, “microglia” emerged as a keyword with strong citation bursts, indicating a growing research focus on neuroinflammation and microglial cell function.

## 4 Discussion

In this study, we used bibliometric analysis to comprehensively review the literature on inflammaging published from 2005 to 2024. Our analysis shows that inflammaging is increasingly becoming a focal point of widespread interest. Over the past 2 decades, the volume of literature has grown significantly, with research articles far outnumbering review articles. This trend suggests increasing complexity in the inflammaging field, as researchers continue to uncover new findings and insights, driving rapid development. Additionally, the growing volume of literature reflects increasing scholarly attention on inflammaging as a key mechanism in the aging process.

The top ten publishing countries include eight from Europe, two from North America, and one from Asia. The United States, with nearly 30% of the total literature, continues to lead in global scientific research. Although Italy’s publication volume is slightly less than that of the United States, it leads in centrality, underscoring its pivotal role in this field. Institutionally, Italy’s significance is confirmed as the University of Bologna, IRCCS INRCA, and Marche Polytechnic University all rank in the top ten, with the University of Bologna leading with 95 papers and a centrality of 0.21. Furthermore, nine of the top ten most prolific authors are Italian, showcasing the outstanding contributions of these researchers and affirming Italy’s significant influence in global inflammaging research. Notably, China, despite being the third highest in publication volume with 187 papers, exhibits a remarkably low centrality of 0.02. This discrepancy may reflect the nature of China’s research contributions, which are substantial in quantity but possibly limited in influence or integration within the broader international research community. Several factors could contribute to this phenomenon, including potential barriers in language and communication, differences in research focus that may not align closely with global trends, or limited international collaborations. Similar challenges are faced by Russia, which, despite having aging populations, uneven resource distribution, and underdeveloped healthcare systems driving a focus on aging-related health issues, has managed to position its Lobachevsky State University of Nizhni Novgorod among the top ten institutions, showcasing active and influential participation in inflammaging research. However, no Chinese institutions made the list, which may reflect limitations in the research environment or international collaboration. International and institutional collaboration in this field remains limited. Therefore, enhancing cross-national and cross-institutional cooperation is crucial for further advancement in inflammaging research.

Journal analysis indicates that inflammaging research primarily focuses on molecular biology/immunology and medical/clinical areas. The top ten journals, such as “Inflammatory Bowel Diseases,” “International Journal of Molecular Sciences,” and “Frontiers in Immunology,” comprise nearly 15% of all publications, underscoring their significant role in this field’s research advancement. Notably, “Nature,” with an impact factor of 50.5, has only one article in this field yet has received 1,062 citations, demonstrating significant impact. The 2019 article “L1 drives IFN in senescent cells and promotes age-associated inflammation” illustrates how LINE-1 (L1) activates type I interferon responses in cellular senescence, crucial for age-related sterile inflammation (De Cecco et al., 2019). This underscores the substantial impact high-quality journal publications can have on the scientific community. Recently, “CELLS-BASEL” has seen a citation burst, with a focus on biomedical applications in cellular research. This surge in citations indicates growing interest in studies that unravel cellular mechanisms of inflammation and aging.

Keyword analysis has revealed hotspots and helped identify eight research themes based on these insights. These themes focus on three primary areas: inflammatory and immune-related diseases, neurological disorders, and interactions between physiological functions and microbial environments. Aging-related inflammation and immune mechanisms are crucial in the development of diseases such as Crohn’s disease and rheumatoid arthritis (Shive and Pandiyan, 2022; Kim and Lee, 2023). The NF-κB signaling pathway, which regulates inflammatory responses, exacerbates the pathology of chronic diseases by upregulating TNF and various interleukins (Singh et al., 2024). Research indicates that therapies targeting the NF-κB pathway, such as small molecule inhibitors and biologics, are significantly effective, underscoring the importance of immune-inflammatory regulation (Zhao et al., 2020). Neurodegenerative disorders like Parkinson’s and Alzheimer’s disease are closely linked to chronic inflammation. For instance, in Parkinson’s disease, excessive microglial cell activation releases inflammatory factors that damage dopaminergic neurons, exacerbating disease progression (Wang T.-F. et al., 2022; Rim et al., 2024; Takahashi and Mashima, 2022). Studies suggest that targeting microglial cell activity or using anti-inflammatory drugs may slow these conditions, indicating the effectiveness of neuroinflammatory regulation as a therapeutic strategy (Scott et al., 2021; Miller and Blanco, 2021). Additionally, the interaction between physiological functions and the microbial environment plays a significant role in inflammaging mechanisms. Particularly, imbalances in the gut microbiome critically influence inflammatory responses during aging and are linked to obesity, diabetes, autoimmune diseases, and aging-related inflammation (Wang Y. et al., 2022; Alsegiani and Shah, 2022). Research shows that modulating gut microbiota composition with probiotics or prebiotics can effectively improve inflammatory states and metabolic health, directly impacting the progression of age-related diseases (Ahmadi et al., 2020; Wang et al., 2021). This keyword research not only deepens our understanding of inflammaging mechanisms but also highlights how these insights translate into clinical treatment strategies.

Burst analysis of keywords highlights the evolving focus of research. Results show a shift from initial focus on specific inflammatory diseases to broader studies on aging and immune modulation. Early studies focused mainly on inflammatory bowel diseases like Crohn’s disease and ulcerative colitis, highlighting inflammaging’s crucial role and the dysregulation of microbial ecosystems in geriatric pathophysiology. Research interest later shifted to the comprehensive mechanisms of immunosenescence, especially those involving chronic inflammatory markers such as C-reactive protein (CRP). As an acute-phase protein, CRP’s elevation is typically linked to inflammatory responses, with its role in aging attracting increasing scrutiny (Kasher et al., 2024; Nejati Bervanlou et al., 2024). Advancements in research techniques have refocused attention on cellular-level exploration, particularly microglial cells. Future research aims to explore methods to alleviate or reverse age-related pathologies by modulating the activity of these cells. In treating neurodegenerative diseases, significant breakthroughs are anticipated through existing medications or new therapeutic developments. This shift highlights the dynamic evolution of inflammaging research from macroscopic clinical observations to microscopic and molecular interventions, potentially impacting medical practices and therapies for aging populations significantly.

## 5 Limitations

Despite the valuable insights from this study, it is important to acknowledge its limitations. Firstly, the research focuses on published academic literature and may not fully account for unpublished or grey literature, potentially limiting a comprehensive understanding of the field. Secondly, the selection of keywords and themes is influenced by subjective preferences, potentially limiting the analysis’s breadth and comprehensiveness. Additionally, the bibliometric analysis results are constrained by the coverage and indexing strategies of the databases used, particularly the WoSCC. The scope of indexed literature and the database’s indexing decisions can affect the comprehensiveness and representativeness of the study, possibly leading to some relevant studies being overlooked. Furthermore, while this study aims to inform clinicians and highlight key topics in the field, the relatively small sample size of included articles may limit the ability to draw broad, definitive conclusions. Future research should expand the scope by including a broader array of databases, unpublished and grey literature, and employing a systematic approach to keyword selection.

## 6 Conclusion

This study presents a comprehensive analysis of development trends and scientific focal points in the field of inflammaging over the past 20 years. The significant growth in literature volume and evolving research hotspots reflect increasing academic interest and the continuous expansion of scientific frontiers in this area. Current research hotspots are centered on inflammatory and immune-related diseases, neurological disorders, and the interactions between physiological functions and microbial environments.

Looking ahead, advances in scientific research and technology are expected to enable future studies to delve deeper into cellular and molecular regulatory mechanisms. Strengthening international and cross-institutional collaboration will also be crucial in accelerating scientific progress. These efforts are expected to drive more impactful translational research, ultimately leading to improved clinical strategies and better health outcomes for aging populations worldwide.

## Data Availability

The original contributions presented in the study are included in the article/supplementary material, further inquiries can be directed to the corresponding authors.
